# Prevention among immigrants: the example of Germany

**DOI:** 10.1186/1471-2458-10-92

**Published:** 2010-02-24

**Authors:** Jacob Spallek, Hajo Zeeb, Oliver Razum

**Affiliations:** 1 University of Bielefeld, Department of Epidemiology & International Public Health, School of Public Health, PO Box 10 01 31, D-33501 Bielefeld, Germany; 2 University Mainz, University Medical Center, Institute of Medical Biostatistics, Epidemiology and Informatics (IMBEI), Department of Epidemiology, Obere Zahlbacher Str. 69, D-55131 Mainz, Germany; 3 University of Bremen, Bremen Institute for Prevention Research and Social Medicine (BIPS), Linzer Straße 10, D-28359 Bremen, Germany

## Abstract

**Background:**

A large and increasing part of the European population has a history of migration. Germany, for example, is home to about 15 million people with migrant background, which amounts to 19% of its population. Migrants may have differences in their lifestyle, health beliefs and risk factors compared to the autochthonous populations.

**Discussion:**

As for example studies on children's participation in routine prevention activities have shown, these differences can have a relevant impact on the access of migrants to the health care system and are likely to lower their participation in prevention programs compared to the autochthonous population. To increase the uptake of prevention programs, barriers to access must be identified and approaches to reduce them must be developed.

**Summary:**

Taking the example of Germany, a need exists for prevention programs that include (migrant sensitive) and specifically address (migrant specific) migrants. These should be of sufficient scale, evidence-based, sustainable and evaluated at regular intervals.

## Background

Migration is a phenomenon affecting all European countries, and having an influence on the health of the migrants as well as of populations of the host countries. Infectious diseases are still relevant in the context of migration. In addition, experiences during travel and in the host countries may negatively affect the health of migrants. Still, migrants largely suffer from similar health problems as the populations of the host countries. In consequence, today's discussion on migrant health is focusing more and more on equity and equality issues. For example, it is being investigated in many countries whether migrants have equal access to health care and social services. Observed health differentials serve as indicators for equity in access. A pertinent question in this context is whether migrants can access preventive services appropriately, and whether they benefit from preventive offers in the same way as the autochthonous population does. This question is understudied, as we show in this article, using the example of Germany.

There are different definitions of the term immigration. According to international definitions, immigration occurs when a person (in this paper always men and women unless otherwise stated) moves his or her centre of living over a socially meaningful distance, and it is international immigration when this occurs across national borders. In this paper, we only address international immigration.

As in several other countries, groups of immigrants in Germany are often defined solely on the basis of nationality and no difference is made between foreigners and immigrants. However, foreign citizenship does not always equate to an immigrant background. For example, there are people with foreign citizenship who were born in Germany and did not migrate themselves, and also immigrants with German citizenship, obtained e.g. through naturalization. In addition, there are people with an ethnic German background whose ancestors had settled in Eastern Europe. Many of these people have now returned to Germany, and are known as 'Spätaussiedler' or 'Aussiedler'.

Also noteworthy is the timing of migration. There can be meaningful differences between immigrants who have newly arrived in the host country, immigrants who have resided in there for years, and immigrants whose parents or grandparents immigrated many decades ago. Immigrant groups are heterogeneous; they include, for example, immigrant workers, their families, contract and seasonal workers, scientists, as well as immigrants with restricted residence permits. In addition, there are the group of refugees and asylum seekers from third world countries with their spouses and families.

Because of Germany's central European location, it has always been both a thoroughfare and an immigrant country. Two major immigration movements occurred since the 1950s, in addition to a large intake of refugees after the Second World War; (i) work migration and immigration of the families of the so called "guest workers", More than half of the 6,74 million people with foreign citizenship currently living in Germany originate from the Mediterranean, the largest portion, 25%, coming from Turkey [1], and (ii) the immigration of ethnic Germans known as Spätaussiedler. In total to date, approximately 4 million Spätaussiedler have migrated to Germany, principally from the former Soviet Union. Of these, about 2.5 million have come since the fall of the Iron Curtain [2].

In the 2005 partial census by the Federal Statistics Department (Mikrozensus), there were a total of 15.3 million people with an immigrant background, which corresponds to about 19% of the German population [3].

Germany has one of the largest immigrant populations in Europe. Due to the heterogeneity of origin and the wide range of reasons for immigration, it is not appropriate to make general statements about 'the' immigrants. Immigrants are a special group - made up of numerous subgroups - that differ to some degree from the majority population. Differences arise due to factors such as the primary reasons for migration, lifestyles (nutrition etc.), or discrimination because of the migrant origin. Immigrants are also more likely to have a below-average income, a lower education, as well as unfavourable working and living conditions [4]. Concerning prevention, there are two core questions: which barriers do these immigrants face regarding access to preventive programs in a highly developed country like Germany and, what has (and could) be done to overcome these barriers?

The health of migrants is determined by a number of factors:

• The conditions in the country of origin before migration (for example, different nutritional exposures, lower quality healthcare, violence, war, torture, and a higher prevalence of certain infectious diseases).

• The conditions during the migration process (for example, psycho-social burdens and stress, hunger, violence, racism, separation from the family).

• The conditions in the host country, both the immediate conditions after arriving (feeling 'foreign', separation from family, racism, language and comprehension problems) and the conditions that can have an influence years or generations later (different cultural and traditional ways of life, racism, lower educational status and social standing, continuing language problems).

In spite of these potentially adverse conditions, it should be noted that immigrants are often highly motivated and open-minded concerning changes, e.g. to adapt to a changed life situation. Through this, and often through to a significant social network, immigrants may have health opportunities alongside their health risks.

European health systems are only slowly adapting to the needs of migrants. For example, immigrant-specific offers in the health system of Germany were for a long time primarily related to infectious disease. During their migration, immigrants have an increased risk of suffering from an infectious disease, which must be taken into account by the health system. 'Exotic' infectious diseases at the population level, however, only constitute a small portion of the overall infectious disease burden and, in addition, are increasingly introduced by returning tourists. A more noteworthy challenge for health services are HIV-positive immigrants coming from high prevalence countries, mainly in sub-Saharan Africa [5]. Other examples include chronic hepatitis infection among African immigrants [6], or tuberculosis, particularly among Spätaussiedler from the former Soviet Union [7]. Infection control laws stipulate that Spätaussiedler and asylum seekers staying in the crowded conditions of refugee accommodation are screened for tuberculosis. However, the specific health situation of immigrants is not only evident in the realm of infectious disease risk, where specific programs for subgroups under risk are sensible. Many health problems of immigrants are similar to the general population, or caused by similar exposures, but the exposures are more or less common in specific immigrant groups resulting in higher or lower incidence or mortality of the immigrants.

For example, an analysis of cause of death statistics shows an overall lower mortality for persons of foreign nationality [8], immigrants with a Turkish background [9], and for the immigrant group of Aussiedler [10]. This advantage levels out over time as mortality rates converge towards those of the German population. This lower mortality is also evident when analysing specific causes of death, such as most cancers and cardiovascular disease [11,12]. Nevertheless, these conditions are the most frequent causes of death both in the autochthonous population and among migrants.

It has often been observed that immigrants have a mortality advantage over the autochthonous population. This phenomenon, known as the "Healthy Migrant Effect", is brought about by the prerequisite of general good health necessary for a prospective migrant labourer who applies for work. A low risk for certain diseases, such as for cardiovascular diseases in the Mediterranean region, may also contribute to this effect. Changes to the "old" risks brought along by the immigrants, and the development of new risks occurring with different lag times, lead to risks that differ from those of the majority population of the host country. For an overview of the current discussion on this topic, see Razum & Twardella 2002 [13] or Spallek & Razum 2008 [14].

In spite of some health advantages, immigrant populations are considered a vulnerable group in terms of health. Immigrant needs should therefore be given proper attention in the field of health research, so that excess risks of specific immigrant groups can be identified and high-risk groups can be targeted. For example, children of immigrants have been found to have a significantly elevated prevalence of dental caries [15,16], and an increased prevalence of obesity [17,18], both in some kind preventable health conditions. Papers by Razum et al. 2008 [19], Zeeb & Razum 2006 [20], and Borde & David 2003 [21] offer deeper insights into epidemiological investigations of immigrant health and healthcare in Germany.

In conclusion, immigrants are a heterogenic population with heterogenic health problems. Some immigrants have specific health problems, e.g. due to infectious diseases, but overall the most frequent health problems of most immigrants are similar to the autochthonous population. This is true especially for immigrants with long duration of stay or for descendants of immigrants.

## Discussion

The goal of prevention for immigrants should be to maintain low risks for diseases such as cancers and conditions related to the cardiovascular system for as long as possible, and to reduce any elevated risks. At the same time, the health system must ensure equitable access to, and effectiveness of, care for immigrants.

Immigrants differ from the majority of the population in terms of their health-related behaviour and use of health care resources. Many have different cultural or traditional ways of life, and frequently a different understanding of sickness and health. This may lead to differences in health-related habits in fields such as nutrition, living and working conditions, as well as in alcohol consumption and smoking [22-24]. The often strong familial coherence or different nutritional habits (e.g. a 'Mediterranean diet'), on the other hand, can lead to a reduced risk for specific diseases with concomitant low use of health care resources.

Differences in the health of migrants relative to that of the majority population could be the expression of differences in health-related habits, other exposures, social deprivation or differing genetic background. Inequalities in health outcomes can besides the influence of these factors serve as indicators for a lack of effective prevention, diagnosis, or adequate treatment of disease. Of particular interest are studies showing differentials in access to health care.

For example, children of Turkish origin appear to have slightly increased risks for non-Hodgkin lymphoma, leukaemia and Hodgkin disease, compared to German children [25] (the causes of these differences need further research). Survival, however, does not differ between Turkish and non-Turkish children with cancer in Germany [26]. This indicates that children of Turkish origin seem to have the same access to the highly standardised diagnosis and treatment of cancer as the majority population.

Another example is maternal mortality, which has been higher among foreign women compared to German women for many years. Recently, however, maternal mortality ratios have been converging [27-29]. This effect is most pronounced for conditions such as haemorrhage early during pregnancy, which requires immediate access to emergency obstetric care. Apparently, access to this type of care is improving.

Infant mortality, however, is still increased among infants of non-German mothers, particularly so among mothers who immigrated to Germany only recently. These figures show that while access to care and uptake of preventive programmes such as ante-natal care are improving, they are still far from optimal; and they fail to fully extend to immigrants who newly arrived in Germany.

Studies on children's participation in routine health screening tests (e.g. for dental problems, general health) also show lower participation rates among immigrant children in Germany [30-32]. Whereas 90% of all German children in the city of Bielefeld had participated in all routine prevention activities up to school age, this proportion was 78% among foreign children born in Germany, and 60% among children born abroad, although the number of cases in this latter group was very small [32]. Similar results have been found for vaccinations. The study from Bielefeld [32] and an analysis of data on primary school pupils in Berlin [18] showed that children of foreign origin have lower immunization rates, or are unable to provide vaccination documents. This shows a clear need for information strategies aimed at immigrants regarding childhood immunization and participation in routine health screening examinations offered by the German health system.

Such differences can be interpreted as a proxy indicator for low participation in prevention programs, and they even persist in immigrants who were born in Germany [33]. There is some evidence that existing prevention programs are less used by immigrants, and sometimes not used at all. However, representative data on participation rates of adult immigrants in screening programs are lacking in Germany.

The low participation of immigrants in prevention programs can partly be attributed to specific access barriers. Such barriers become apparent in different ways depending on the background, cultural and religious origins, length of residency in Germany, language abilities, gender, and education and social status of the immigrant group concerned.

For example, the differences between the health system of Germany and the country of origin pose problems to immigrants. They find it difficult to obtain knowledge of available prevention programs and to identify appropriate entry points into the system. The problem may lie with a lack of German language skills, which makes direct communication with medical staff more difficult. Differences in concepts underlying the understanding of health and disease, for example among Muslim patients [34], can lead to a lower or less effective use of health services. Some immigrants have a more pain-oriented depiction of symptoms [35] which can mislead doctors to a false perception of the underlying disease, and thus to an incorrect diagnosis and a wrong decision concerning what treatment should be given. In addition, immigrants from different cultural backgrounds perceive aspects of disease as an established part of spirituality, morality or religion, while most patients of Western European origin would perhaps regard these aspects as "purely" medical. When the health system rests on a strong natural sciences and medico-physical foundation, as is the case in many European countries including Germany, immigrants may perceive particular obstacles towards accessing health and preventive care [33].

Besides these barriers, a low social status may lead to a low utilization of the healthcare system, including preventive services. One of the contributing factors may be the fee or co-payment required for GP and specialist services, even though most preventive care is exempt. This is not uniquely an immigrant problem; rather it is a general problem of lower socioeconomic groups (in which immigrants are over-represented) and should be addressed by prevention programs in general.

## Summary

### Towards an Immigrant-Focussed and Immigrant-Sensitive Prevention

Some immigrants have specific health problems, requiring specific prevention programs. Access to existing prevention programs is lower in the immigrant population, due to various barriers, which points out a need for a higher migrant sensitiveness of prevention programs for the general population.

In Germany there is an increasing number of research projects related to immigrant health. They are diverse in topic and approach, so it is difficult to describe them in general terms. The database of the *Bundeszentrale für gesundheitliche Aufklärung *(Federal Department for Health Education) offers an overview of these projects [36]. More then 2600 prevention projects are listed, of which approximately 25% address immigrants (or claim to be migrant sensitive). However, only 6% are specifically aimed at this group (migrant specific). Many of these projects are conducted by local associations, charitable organizations, or other agencies, so they usually have constraints in terms of geographic coverage or length of funding period. Less than 5% of these projects have been evaluated externally, and evaluation mostly focuses on process rather than outcome, so it is currently not possible to assess their preventive effectiveness [37].

Offering written information in a non-German language is now becoming more common in the German health system, thus fixing the language barrier. However, this is often limited to translations into the language of the largest immigrant groups, such as Turkish and Russian. However, it is not only important to translate available information material; it should also be tailored to the needs and the cultural background of immigrants. This requires extra efforts beyond translation. In addition, visual communication material for illiterate immigrants with German language problems is rarely available.

The development and establishment of special communication and information channels for immigrants has been neglected by the German health system. Such channels are important to reduce access barriers, in particular for immigrants with a greater "distance" between own and host-country culture. e.g. barriers for Muslim women to participate in breast cancer screening [38,39]. Furthermore, migrants are heterogeneous with regard to cultural and traditional backgrounds - even within one ethnic group or country of origin. Generalized solutions are therefore unlikely to work.

One strategy to solve the problem of lower access of immigrants due to language or cultural barriers is to train mediators with migrant background who disseminate information on health topics within their own cultural group, and facilitate access to the health system. An example of this approach is the project "*Mit Migranten für Migranten*" (which literally translates to "With Migrants for Migrants"), (MIMI; http://www.bkk-promig.de/) developed by the Ethno-Medical Centre in Hannover with the support of a health insurance company. Trained mediators of different ethnicities organize informational events on health and prevention topics for people of their own cultural background. An evaluation of this project shows promising results [37]. This is an example of health promotion among immigrants using mediators. By now, the project is operating in 24 regions in Germany. There is now a need for formal outcome evaluation before programs using mediators with migration background can be recommended on a broader scale.

Another example of successful health promotion (this one without mediators with a migration background) among immigrants in Germany is a project run by the Non-Governmental Organisation *pro familia*. *Pro familia *is an advisory body for families. An evaluation showed that the uptake of their information was low among immigrant women. So *pro familia *developed an outreach approach, offering information on reproductive health and STDs for women, e.g. in German classes for foreigners, in Kindergartens and in cafés [40].

The strength of these tailored programs is their ability to adapt interventions to the specific needs of subgroups, e.g. dissemination of information about reproductive health for Muslim women with poor knowledge of the German language. A disadvantage of specific programs is that they need a comparably large amount of resources, in particular money, personnel, and time.

For this reason there is a need to adapt existing, and to develop new, prevention programs addressing the general population in such a way that they reach immigrants as well. This is of particular importance as the major health problems of immigrants are similar to those of the autochthonous population. A strategy to reach sustainable improvements in the German and in other European health systems can be diversity management. Its goal is to reduce access barriers due to social or cultural differences through continuous consideration (mainstreaming) of diversity (of which migration background is only one example) in all processes and activities. Geiger [41] provides an in introduction to this concept. A core aspect is to create awareness among doctors and other healthcare providers of the diverse needs of all their patients, in this case of the specific situations and needs of migrants. Diversity Management is not restricted to offering doctors continuing education to better meet the needs of their patients in their daily work; rather it is related to all levels and players of the health system. In future, an increasing part of health workers and planners in Germany will have a migration background. While they also will need to be trained in Diversity Management, their knowledge of a second culture and language may actually help them to deal with the needs of immigrant patients.

Strategically organising the health system to better accommodate the needs of immigrants is a complex process with different possible approaches. This can be described in terms of two extremes. The first extreme is that the immigrants are expected to adapt to the existing health system. The immigrants will be enabled to take advantage of existing preventive programs through information, education and empowerment, ultimately to the same extent as the general population, without changing the programs themselves. In the second extreme, separate preventive activities are developed and targeted at immigrants. These two approaches are often termed immigrant-sensitive (integrating and mainstream) and immigrant-specific (separating, positively discriminating). The advantages of an immigrant-specific approach are its adaptability to specific situations and a quick implementation of changes. The advantages of immigrant-sensitive offers lie in their broad coverage of a wide spectrum of needs (and therefore potential savings in costs), sustainability, integrating effects and ability to consider different and multiple needs and cases at once. Immigrant-specific offers could probably be made only for the largest or highest risk groups, and would therefore exclude a variety of other immigrant groups. We propose diversity management as the most appropriate "third" way. Diversity management offers a better regard for the specific needs of immigrants in all the health sectors' processes and activities. If required, short-term programs complimenting general diversity mainstreaming, can be implemented, addressing specific subgroups within immigrant communities, such as elderly immigrants, women, children and adolescents.

In addition, there is a need for more research on immigrant health. This includes methodological aspects such as addressing the lack of definitions and importance of immigrant backgrounds in the health information systems. As prevention and public health develop towards more evidence-based approaches, migrants need to be routinely included in studies of intervention effects, and specific interventions tailored to their needs should be evaluated routinely.

Results of such studies, coupled with best-practice experiences, will be important for an improved uptake of and access to preventive services by immigrants in Germany and beyond, and thus ultimately for more equitable health care systems in Europe.

## Competing interests

The authors declare that they have no competing interests.

## Authors' contributions

JS drafted the manuscript. JS and OR conceived the work. JS, HZ and OR conducted the literature overview. JS, HZ and OR contributed to the discussion and debate. All authors read and approved the final manuscript.

## Pre-publication history

The pre-publication history for this paper can be accessed here:

http://www.biomedcentral.com/1471-2458/10/92/prepub
